# Bickerstaff encephalitis in childhood: a review of 74 cases in the literature from 1951 to today

**DOI:** 10.3389/fneur.2024.1387505

**Published:** 2024-03-12

**Authors:** Luca Gregorio Giaccari, Donatella Mastria, Rosella Barbieri, Rossella De Maglio, Francesca Madaro, Gianfranco Paiano, Maria Caterina Pace, Pasquale Sansone, Giuseppe Pulito, Luciana Mascia

**Affiliations:** ^1^Department of Anesthesia and Intensive Care, “Vito Fazzi” Hospital, Lecce, Italy; ^2^Department of Women, Child, General and Specialist Surgery, University of Campania “L. Vanvitelli”, Naples, Italy; ^3^Department of Experimental Medicine, University of Salento, Lecce, Italy

**Keywords:** Bickerstaff brainstem encephalitis, Bickerstaff’s encephalitis, Bickerstaff’s syndrome, children, pediatric

## Abstract

Bickerstaff brainstem encephalitis (BBE) is a rare autoimmune disease characterized by the subacute onset of bilateral external ophthalmoplegia, ataxia, and decreased level of consciousness. BBE is part of a group of rare autoimmune diseases in children that can affect the nervous system at any level. The onset of neurological deficits is often sudden and nonspecific. The diagnosis is based on clinical findings and abnormal findings on cerebrospinal fluid (CSF), electroencephalography (EEG), electromyography (EMG), and magnetic resonance imaging (MRI). BBE is associated with the presence of the antiganglioside antibody, anti-GQ1b and anti-GM1. Intravenous immunoglobulin (IVIg) and plasma exchange are often used as treatments for these patients. We conducted a review on clinical presentation, diagnosis, treatment and outcome of reported cases of BBE. 74 cases are reported in the literature from the first cases described in 1951 to today. The prevalence is unknown while the incidence is higher in males. In 50% of cases, BBE occurs following respiratory or gastrointestinal tract infections. The most frequent initial symptoms were consciousness disturbance, headache, vomiting, diplopia, gait disturbance, dysarthria and fever. During illness course, almost all the patients developed consciousness disturbance, external ophthalmoplegia, and ataxia. Lumbar puncture showed pleocytosis or cytoalbuminological dissociation. Abnormal EEG and MRI studies revealed abnormalities in most cases. Anti-GQ1b antibodies were detected in more than half of the patients; anti-GM1 antibodies were detected in almost 40% of patients. Treatment guidelines are missing. In our analysis, steroids and IVIg were administered alone or in combination; as last option, plasmapheresis was used. BBE has a good prognosis and recovery in childhood is faster than in adulthood; 70% of patients reported no sequelae in our analysis. Future studies need to investigate pathogenesis and possible triggers, and therapeutic possibilities.

## Introduction

Bickerstaff brainstem encephalitis (BBE) was first described by Bickerstaff and Cloake in 1951 under the title “Mesencephalitis and rhombencephalitis” (1). A few years later, Bickerstaff named this condition “brainstem encephalitis” (2).

BBE is a rare autoimmune disease characterized by the subacute onset of bilateral external ophthalmoplegia, ataxia, and decreased level of consciousness (3). Pupillary abnormalities, bilateral facial paralysis, Babinski’s sign, and bulbar paralysis are commonly present (4). Presence of limb weakness indicates overlap with Guillain–Barré syndrome (GBS) (3, 4).

The prevalence is unknown. According to a Japanese nationwide survey, the annual incidence of BBE is estimated to be approximately 0.078 per 100,000 inhabitants (5). The incidence of BBE is higher in males (male/female ratio 1.3) with an average age at onset of 39 years (5).

BBE has been reported to occur often after upper respiratory or gastrointestinal tract infections (6, 7). Although the exact pathological mechanism is not completely understood, BBE is associated with the presence of the antiganglioside antibody, anti-GQ1b. These antibodies are highly specific for patients with BBE and also GBS, Miller Fisher syndrome (MFS), and external ophthalmoplegia (8); anti-GQ1b antibodies are present in 68% of patients with BBE (9). Anti-GQ1b antibody testing are not necessary for a definitive diagnosis of BEE (3). The detection of these antibodies, however, is useful to confirm the diagnosis of BBE when incomplete syndromes or atypical symptoms are present, or when an altered mental status does not allow the evaluation of ataxia.

Clinical features include a classic triad of ataxia, ophthalmoplegia and altered consciousness (10). Other common features include hyperreflexia, Babinski’s sign, deep sensory impairment, facial weakness, bulbar palsy and nystagmus (10).

Despite the diagnosis being based on clinical findings, abnormal findings on cerebrospinal fluid (CSF), electroencephalography (EEG), electromyography (EMG), and magnetic resonance imaging (MRI) are common. CSF analysis often shows evidence of albumin-cytologic dissociation and pleocytosis (9, 10). At first, albumin-cytologic dissociation occurred in 25% of the BBE and pleocytosis in 32% of the BBE; during the second week, albumin-cytologic dissociation of CSF occurred in 46% of the BBE patients and pleocytosis in 31% of the BBE (9). EEG and EMG are indicative of central nervous system (CNS) impairment and predominantly in the brainstem (9). Patients with BBE showed a characteristic “unarousable sleep-like” EEG (11). In about one-third of the BBE patients, MRI shows high-intensity areas on T2-weighted images of the brainstem, thalamus, cerebellum and cerebrum (12).

There is a lack of consensus on the management of the BBE (13). Intravenous immunoglobulin (IVIg) and plasma exchange are often used as treatments for these patients (13).

The BBE course is generally monophasic with complete remission of symptoms within 6 months in over half of the patients.

Very few studies investigated pediatric BBE and its incidence rate is unknown (14–16). BBE is part of a group of rare autoimmune diseases in children that can affect the central or peripheral nervous system at any level (17, 18).

### Aims

We conducted a review on clinical presentation, diagnosis, treatment and outcome of reported cases of Bickerstaff brainsteam encephalitis. To the best of our knowledge, this review is one of the first to address the BBE in childhood. We believe that clinical cases and case series could contribute relevant knowledge that should be considered in this review, especially when data from randomized and observational studies are not available or insufficient.

## Materials and methods

### Protocol and literature search strategy

We conducted a narrative review. Narrative reviews are useful educational articles because they provide a broad perspective on a topic and often describe the history or development of a problem or its management (19).

The main electronic databases (PubMed, Embase, Scopus, Google Scholar and Cochrane Library) were screened to identify studies reporting Bickerstaff brainstem encephalitis cases in children and adolescents. We used a combination of keywords (MESH terms) including “Bickerstaff brainstem encephalitis,” “Bickerstaff’s encephalitis,” “Bickerstaff’s syndrome,” “children” or “pediatric.” Only English articles were included.

The initial search was performed on January 15, 2024. All articles were included from the first described case made by Bickerstaff in 1951 to the end of December 2023.

### Eligibility criteria

The population, intervention, comparison, and outcome (PICO) criteria were applied to the research question (see Table 1). Patients younger than 18 years diagnosed with Bickerstaff brainstem encephalitis were considered as the population (P); the intervention (I) was the diagnostic and therapeutic management; the comparison (C) concept was not applicable to the research question; length of hospitalization and the presence of sequelae were considered the outcomes (O) for this systematic review.

**Table 1:** PICO criteria for including studies.

Population	Patients younger than 18 years diagnosed with Bickerstaff brainstem encephalitis.
Intervention	Diagnostic and therapeutic management.
Comparator	No comparator.
Outcomes	Length of hospitalization, presence of sequelae.
Study tipe	Case report, observational study, clinical trial, randomized clinical trial.
Time	From 1951 to today.

Diagnostic criteria were based on those published by Wakerley et al. for BBE and BBE with overlapping GBS (3).

Studies with incomplete outcomes were excluded. Studies written not in English were excluded from this review.

### Outcomes

The outcome was to analyze BEE in childhood focusing on clinical presentation, diagnosis, treatment and outcome. We wanted to evaluated the length of hospitalization and the incidence of sequelae.

### Selection of studies

Titles and abstracts were screened by two researchers (LGG and DM) to identify keywords. We eliminated studies that clearly did not satisfy PICO criteria and obtained full copies of the remaining studies. The selected articles were read in full by the two independent reviewers and, in case of disagreement, a third reviewer (LM) was consulted.

### Data extraction and management

Data extracted included the following: age and sex of participants; number of participants enrolled and completing the study; preinfectious history; symptoms and signs of BBE; diagnostic management including CSF analysis, computed tomography (CT) and/or MRI examination, EEG, EMG, and serum antibody testing; therapy management; outcomes in terms of length of hospitalization and sequelae.

### Data analysis

Data were analyzed using standard computer program (Excel, 2016). Symptoms and signs are reported as a ratio between the number of patients in which the variable was present (n) and the total number of patients (N): n/N (%). We assumed that they were absent rather than missing if they were not cited in the manuscript, to account for reporting bias, and therefore described as zero (n) out of the total number of reported cases (N). Diagnostic studies are reported as a ratio between the number of positive studies (n) and the total number of performed studies (N): n/N (%). Other data are reported as mean ± standard deviation (SD).

## Results

We included 41 studies (20–60), for a total of 74 cases. 73% of cases were among males (54 males, 20 females). The mean age at onset was 8.65 ± 4.93 years (7 months – 18 years).

### Preinfection history

In half of the cases, a previous illness was reported 10.12 ± 6.99 days before the onset of neurological symptoms. As shown in Table 2, upper respiratory tract infections (URIs) occurred in 17 cases and lower respiratory tract infections (LRIs) in 10 cases. Gastroenteritis and diarrhea were reported in 8 patients, while other unspecified infections were reported in 4 patients. Twenty-three cases were not associated with previous disease.

**Table 2:** Preinfection history before BBE onset.

	No
Previous disease - Upper respiratory infection - Lower respiratory infection - Gastroenteritis/Diarrehoea - Others - None	37 17 10 8 4 23
Agents - *M. pneumoniae* - *H. influenzae* - *C. jejuni* - CMV - *Salmonella typhi* - Influenza type B virus - SARS-CoV-2	14 5 2 3 1 1 1 1
Days before (days)	10.12 ± 6.99

When a microbiological study was performed, the most frequently diagnosed microbiological agents were *M. pneumoniae* (*n* = 5), *C. jejuni* (*n* = 3) and *H. influenzae* (*n* = 2). One case of BBE occurred 2 weeks after COVID-19 vaccination (40).

### Symptoms and signs

At the onset of BBE, patients presented an altered state of consciousness in 46% of cases, 5 of which were due to seizures (see Table 3). Headache and vomiting were present in 21 and 15 patients, respectively. Sixteen patients had diplopia and 12 were dysarthric. Limb weakness was found in 9 patients; gait disturbances were found in 23 patients. A febrile state was present in 40.5% of cases at initial presentation.

**Table 3:** Initial symptoms and neurological signs during BBE course.

Initial symptoms	No. (%)
- Consciousness disturbance - Seizure	35 (47.3) 5 (6.8)
- Headache	21 (28.4)
- Vomiting	15 (20.3)
- Diplopia	16 (21.6)
- Strabismus	3 (4.1)
- Blepharoptosis	2 (2.7)
- Limb weakness	9 (12.2)
- Gait disturbance	23 (31.1)
- Dysarthria	12 (16.2)
- Fever	30 (40.5)
Neurological sign during illness course	
- Consciousness disturbance	40 (54.1)
- Ophthalmoplegia	51 (68.9)
- Ocular motor weakness and ptosis (n. III, IV, VI)	36 (48.6)
- Facial weakness (n. VII)	42 (56.8)
- Bulbar weakness (n. IX, X, XI)	39 (52.7)
- Mild limb weakness	27 (36.5)
- Muscle stretch reflex - Absent or decreased - – Normal or brisk	42 (56.8) 28 (37.8) 14 (19.0)
- Babinski sign	18 (24.3)
- Ataxia	46 (62.2)
- Sensory disturbance	4 (5.4)
- Autonomic instability	6 (8.1)

As shown in Table 2 at initial evaluation, disturbance of consciousness affected more than 50% of patients. Fifty-one cases of ophthalmoplegia were detected. Neurological examination of the cranial nerves showed ocular motor weakness and ptosis (*n* = 36), facial weakness (*n* = 42), and bulbar weakness (*n* = 39). Twenty-seven patients presented with limb weakness; 62.2% of patients were ataxic. Muscle reflex abnormalities were present in 56.8% of patients: in 28 cases they were absent or decreased, while in 14 cases they were brisk. Babinski sign was elicited in 18 patients. 8.1% of patients showed dysautomia, while 5.4% had sensory disturbances.

### Diagnostics

In more than 90% of cases the diagnostic assessment included performing a lumbar puncture. As shown in Table 4, 55.1% of all were pathological: pleocytosis was reported 15 times and cytoalbuminological dissociation 23 times. Abnormal EEG was reported in more than 85% of those performed. Eleven patients had abnormal EMG. MRI studies were performed in 78.4% of patients, showing abnormalities in 24 cases. When CT studies were performed, no abnormalities were revealed.

**Table 4:** Diagnostic assessment in BBE.

Neurodiagnostic studies	No./Tot. (%)
- Abnormal LP - CSF pleocytosis - CSF cytoalbuminological dissociation	38/69 (55.1) 15/69 (21.7) 23/69 (33.3)
- Abnormal EEG	29/34 (85.3)
- Abnormal MRI	24/58 (41.4)
- Abnormal CT	0/9 (0)
- Abnormal EMG	11/21 (52.4)
Serum antibodies	
- anti-GQ1b +	24/44 (54.5)
- anti-GM1 +	3/8 (37.5)

CT, computed tomography; EEG, electroencephalography; EMG, electromyography; LP, lumbar puncture; MRI, magnetic resonance imaging.

Serum antibody samples were collected in 46 cases. In 54.5% of these cases, anti-GQ1b antibodies were found positive. Anti-GM1 antibodies were positive in 3 of 8 cases.

### Therapy

In more than 83% of patients, one or more treatments were started. Steroids were administered 10 times, IVIG 11 times, and a combination of steroids and IVIG 18 times. Plasmapheresis was performed for 4 patients. Antibiotics (*n* = 16) and antivirals (*n* = 15) were also administered.

Intensive Care hospitalizations was necessary for 16 patients. Fourteen required mechanical ventilation.

The median length of stay in ICU was 12.85 ± 7.44 days.

This is shown in Table 5.

**Table 5:** Therapy management in BBE.

	No
Treatment - Steroids - IVIg - Steroids + IVIg - Plasmapheresis - Antibiotics - Antiviral	10 11 18 4 16 15
Intensive Care Hospitalization - Mechanical ventilation - Tracheostomy - EVD ICU LOS (days)	16 14 2 2 12.85 ± 7.44

EVD, external ventricular drainage; ICU LOS, Intensive Care Unit length of stay; IVIg, intravenous immunoglobulin.

### Outcome

As shown in Table 6, the length of hospitalization was 24.2 ± 13.75 days. For 66.2% of patients, outcome was reported. After 4.2 ± 2.56 months, 34 patients had no sequelae, 13 had sequelae and 2 died.

**Table 6:** Outcome in BBE.

LOS Hospitalization (days)	24.2 ± 13.75
Sequelae None Any Died	34 13 2

LOS, length of stay.

## Discussion

According to the literature (14–18), BBE is quite uncommon especially in children. The onset of neurologic deficits is often sudden, although nonspecific symptoms may precede neurologic manifestations by several weeks. Diagnosis by MRI and CSF analysis is essential in children when autoimmune encephalitis is suspected. The central diagnostic test is the search for neural antibodies. There is no difference in the therapeutic approach to children/adolescents and adults.

Our review provides a case analysis of BEE in childhood focusing on clinical presentation, diagnosis, treatment and outcome.

The prevalence is unknown while the incidence is higher in males. Our analysis showed a 3:1 male to female ratio, as in previous studies (15, 16). This is in contrast to general data that females are more susceptible than males to a variety of autoimmune diseases (61).

In 50% of cases an infection is reported in the 2 weeks before the onset of neurological symptoms. BBE occurs following respiratory or gastrointestinal tract infections, most frequently caused by *M. pneumoniae, H. influenzae*, and *C. jejuni*. A case study reported on an episode of BBE following COVID-19 vaccination (41). SARS-CoV-2 infection is associated with other neurological condition, such as Guillain-Barrè syndrome (62) and Miller Fisher syndrome (63). The etiology is unfortunately unknown, as for other neurological syndromes (64). A transient autoimmune response to myelin or other self-antigens through molecular mimicry during the infectious episode preceding symptoms, leading to destruction of the cerebral white matter, spinal cord, and optic nerves causing demyelination (64).

BBE is usually a monophasic disease; only one case of recurrent BBE is reported (24). The most frequent initial symptoms were consciousness disturbance, headache, vomiting, diplopia, gait disturbance, dysarthria and fever. During illness course, almost all the patients developed consciousness disturbance, external ophthalmoplegia, and ataxia. Ocular motor weakness and ptosis, facial weakness, and bulbar weakness were common manifestations. Extensor plantar response (Babinki’s sign) was present. Deep tendon reflexes were usually absent or decreased, but can be normal or brisk. Sensory disturbance and autonomic instability may occur.

When atypical initial symptoms such as symmetrical flaccid limb weakness and dysesthesias occur, these cases were classified as BBE with overlapping/coexisting GBS (25, 31, 40, 47).

Lumbar puncture is a high-priority test in cases of suspected central nervous system disease (65). In our analysis, an abnormal lumbar puncture was found in more than half of the patients; the main CSF alterations found were albuminocytological dissociation (33.3%) and pleocytosis (21.7%). Albuminocytological dissociation occurs during the first week of illness in 25% of cases and increases up to 46% of cases in the second week; 32% of the BBE patients show CSF pleocytosis (9). These CSF findings prove an inflammatory origin of neurological disturbances compatible with autoimmune encephalitis, such as BBE (66).

EEG abnormalities were reported in more than 85% of cases in which the exam was performed. The frequency of abnormal EEG findings indicated CNS involvement, consistent with disturbance of consciousness. EEG changes correlate with the level of consciousness in patients with BBE and often show predominant N1 and/or N2 sleep patterns, even with external stimuli (“unarousable sleep-like” EEG), including the fusiform coma pattern (11). The deterioration of consciousness in BBE may be caused by a dysfunction of the brainstem reticular formation (11).

Abnormal EMG was found in more than 50% of patients in this analysis. Electrophysiologically studies showed reduced or non-recordable compound muscle action potentials (CMAPs) and sensory nerve action potentials (SNAPs). This is evidence of demyelination or axonal degeneration and is found in half of cases (9, 12).

CT scans showed no abnormalities. MRI is the gold standard technique for brain imaging in encephalitis (67). Abnormal MRI lesions, such as high-intensity areas on T2-weighted images of the brainstem, thalamus, cerebellum and cerebrum, were present in BBE patients (12). This alteration corresponds to vasogenic edema and may be reversible and not visible on MRI. When performed, MRI showed abnormalities in 41.4% of cases.

Anti-GQ1b antibodies were detected in more than half of the patients. The oculomotor (III), trochlear (IV), and abducens (VI) nerves, muscle spindles in the limbs, and the reticular formation in the brainstem have significantly higher percentages of GQ1b (8, 68). Infection by microorganisms bearing the GQ1b epitope can induce the production of anti-GQ1b immunoglobulin G (IgG) antibodies in susceptible patients. Anti-GQ1b antibodies bind to GQ1b antigens expressed on cranial nerves and muscle spindles inducing FS. It is possible that in some cases the antiGQ1b antibodies reach the brainstem and bind to GQ1b, inducing BBE (8, 68).

The presence of anti-GM1 antibodies was detected in almost 40% of patients. The role of antiganglioside antibodies in the pathogenesis of BBE as well as other diseases is not completely understood. They could participate in direct damage to the structure they bind to, be a consequence of several types of infectious diseases, or facilitate many immune-mediated pathological mechanisms (7).

In the included studies, the diagnosis of BBE was not based only on the symptoms, which were not always typical (external ophthalmoplegia, ataxia, disturbance of consciousness). In our analysis, typical MRI images (high signal lesion on T-2-weighted images located in the brainstem) and high titers of anti-GQ1b antibodies may contribute to the diagnosis of BBE.

There are no established guidelines for treatment and currently different regimens are used based on the patient’s clinical status and the doctor’s opinion. Autoimmune encephalitis, including BBE, responds to immunomodulatory therapy such as IVIG and plasmapheresis (69). This treatment is accompanied by the administration of intravenous corticosteroids (69). In our analysis, steroids and intravenous immunoglobulins were administered alone or in combination. As last option, plasmapheresis was used.

Among corticosteroids, methylprednisolone is commonly used at a dose of 1 g per day for 3–5 days followed by oral prednisone 1 mg/kg/day for 1–4 weeks (64, 69). IVIG has many immunomodulatory and anti-inflammatory effects; it is used for many neurological disorders. The IVIG doses range is between 400 mg/kg/day for 5 days or a more rapid course of 1–2 g/kg given over 1–2 days (64, 70). Plasmapheresis is a reasonable option to consider in BBE patients with no or limited improvement after corticosteroids or IVIG (71). Normally 1 session is performed every other day for 5–7 cycles (30, 64). Patients included in this review did not require the use of second-line agents, such as rituximab or cyclophosphamide.

The length of hospitalization was approximately 24 days. More than 20% of patients required ICU admission for an average of approximately 13 days, mainly due to depressed level of consciousness and respiratory insufficiency. Almost all of them required mechanical ventilation. These rates are lower compared to the adult population with autoimmune encephalitis; in an observational study, 57% of patients were intubated for an average of approximately 1 month and 68% of them required tracheostomy (72). No particular characteristics were identified in BBE cases requiring ICU admission.

BBE has a good prognosis and recovery in childhood is faster than in adulthood, usually resolving within 4 to 6 weeks after starting treatment (14, 16). Concerning the outcome, 70% of patients reported no sequelae in our analysis. The death rate was 2.7% (24, 59). Death is a very rare outcome in patients with BBE receiving optimal treatment.

### Limitations

This review presents some limitations. First, it was based on case series and case reports. They are often excluded from systematic reviews due to the greater potential for bias, especially severity bias and selection bias. In this report, these studies contribute to the available evidence base, and their results supplement the limited evidence available from other studies. Second, a meta-analysis was not performed, due to the design of most of the studies (case report, case series) and the lack of a comparator.

## Conclusion

BBE is a rare autoimmune disease, even in childhood. Its knowledge is essential for the early recognition of the patient suffering from BBE and therefore for its management. The pathogenesis is unclear. The combination of clinical, radiological and laboratory data is the basis of a correct diagnosis. There are few indications for correct treatment which is mainly based on immunomodulatory therapy including IVIG and in the most serious cases on plasmapheresis. Fortunately, the prognosis is good, with a good recovery rate. Future studies need to investigate the pathogenesis, possible triggers and therapeutic possibilities.

## Author contributions

LG: Conceptualization, Data curation, Formal analysis, Investigation, Methodology, Project administration, Resources, Software, Validation, Visualization, Writing – original draft, Writing – review & editing. DM: Supervision, Writing – review & editing. RB: Data curation, Formal analysis, Methodology, Writing – review & editing. RM: Data curation, Formal analysis, Methodology, Writing – review & editing. FM: Data curation, Formal analysis, Methodology, Writing – review & editing. GPa: Data curation, Formal analysis, Methodology, Writing – review & editing. MP: Data curation, Formal analysis, Methodology, Writing – review & editing. PS: Data curation, Formal analysis, Methodology, Writing – review & editing. GPu: Supervision, Validation, Writing – review & editing. LM: Supervision, Validation, Writing – review & editing.
